# Crime forecasting: a machine learning and computer vision approach to crime prediction and prevention

**DOI:** 10.1186/s42492-021-00075-z

**Published:** 2021-04-29

**Authors:** Neil Shah, Nandish Bhagat, Manan Shah

**Affiliations:** 1 Department of Computer Engineering, Sal Institute of Technology and Engineering Research, Ahmedabad, Gujarat 380060 India; 2 Department of Chemical Engineering, School of Technology, Pandit Deendayal Energy University, Gandhinagar, Gujarat 382426 India

**Keywords:** Machine learning, Computer vision, Crime forecasting

## Abstract

A crime is a deliberate act that can cause physical or psychological harm, as well as property damage or loss, and can lead to punishment by a state or other authority according to the severity of the crime. The number and forms of criminal activities are increasing at an alarming rate, forcing agencies to develop efficient methods to take preventive measures. In the current scenario of rapidly increasing crime, traditional crime-solving techniques are unable to deliver results, being slow paced and less efficient. Thus, if we can come up with ways to predict crime, in detail, before it occurs, or come up with a “machine” that can assist police officers, it would lift the burden of police and help in preventing crimes. To achieve this, we suggest including machine learning (ML) and computer vision algorithms and techniques. In this paper, we describe the results of certain cases where such approaches were used, and which motivated us to pursue further research in this field. The main reason for the change in crime detection and prevention lies in the before and after statistical observations of the authorities using such techniques. The sole purpose of this study is to determine how a combination of ML and computer vision can be used by law agencies or authorities to detect, prevent, and solve crimes at a much more accurate and faster rate. In summary, ML and computer vision techniques can bring about an evolution in law agencies.

## Introduction

Computer vision is a branch of artificial intelligence that trains the computer to understand and comprehend the visual world, and by doing so, creates a sense of understanding of a machine’s surroundings [1, 2]. It mainly analyzes data of the surroundings from a camera, and thus its applications are significant. It can be used for face recognition, number plate recognition, augmented and mixed realities, location determination, and identifying objects [3]. Research is currently being conducted on the formation of mathematical techniques to recover and make it possible for computers to comprehend 3D images. Obtaining the 3D visuals of an object helps us with object detection, pedestrian detection, face recognition, Eigenfaces active appearance and 3D shape models, personal photo collections, instance recognition, geometric alignment, large databases, location recognition, category recognition, bag of words, part-based models, recognition with segmentation, intelligent photo editing, context and scene understanding, and large image collection and learning, image searches, recognition databases, and test sets. These are only basic applications, and each category mentioned above can be further explored. In ref. [4], VLFeat is introduced, which is a library of computer vision algorithms that can be used to conduct fast prototyping in computer vision research, thus enabling a tool to obtain computer vision results much faster than anticipated. Considering face detection/human recognition [5], human posture can also be recognized. Thus, computer vision is extremely attractive for visualizing the world around us.

Machine learning (ML) is an application that provides a system with the ability to learn and improve automatically from past experiences without being explicitly programmed [6–8]. After viewing the data, an exact pattern or information cannot always be determined [9–11]. In such cases, ML is applied to interpret the exact pattern and information [12, 13]. ML pushes forward the idea that, by providing a machine with access to the right data, the machine can learn and solve both complex mathematical problems and some specific problems [14–17]. In general, ML is categorized into two parts: (1) supervised ML and (2) unsupervised ML [18, 19]. In supervised learning, the machine is trained on the basis of a predefined set of training examples, which facilitates its capability to obtain precise and accurate conclusions when new data are given [20, 21]. In unsupervised learning, the machine is given a set of data, and it must find some common patterns and relationships between the data its own [22, 23]. Neural networks, which are important tools used in supervised learning, have been studied since the 1980s [24, 25]. In ref. [26], the author suggested that different aspects are needed to obtain an exit from nondeterministic polynomial (NP)-completeness, and architectural constraints are insufficient. However, in ref. [27], it was proved that NP-completeness problems can be extended to neural networks using sigmoid functions. Although such research has attempted to demonstrate the various aspects of new ML approaches, how accurate are the results [28–30]?

Although various crimes and their underlying nature seem to be unpredictable, how unforeseeable are they? In ref. [31], the authors pointed out that as society and the economy results in new types of crimes, the need for a prediction system has grown. In ref. [32], crime trends and prediction technology called Mahanolobis and a dynamic time wrapping technique are given, delivering the possibility of predicting crime and apprehending the actual culprit. As described in ref. [33], in 1998, the United States National Institute of Justice granted five grants for crime forecasting as an extension to crime mapping. Applications of crime forecasting are currently being used by law enforcement in the United States, the United Kingdom, the Netherlands, Germany, and Switzerland [34]. Nowadays, criminal intellect with the help of advances in technology is improving with each passing year. Consequently, it has become necessary for us to provide the police department and the government with the means of a new and powerful machine (a set of programs) that can help them in their process of solving crimes. The main aim of crime forecasting is to predict crimes before they occur, and thus, the importance of using crime forecasting methods is extremely clear. Furthermore, the prediction of crimes can sometimes be crucial because it may potentially save the life of a victim, prevent lifelong trauma, and avoid damage to private property. It may even be used to predict possible terrorist crimes and activities. Finally, if we implement predictive policing with a considerable level of accuracy, governments can apply other primary resources such as police manpower, detectives, and funds in other fields of crime solving, thereby curbing the problem of crime with double the power.

In this paper, we aim to make an impact by using both ML algorithms and computer vision methods to predict both the nature of a crime and possibly pinpoint a culprit. Beforehand, we questioned whether the nature of the crime was predictable. Although it might seem impossible from the outside, categorizing every aspect of a crime is quite possible. We have all heard that every criminal has a motive. That is, if we use motive as a judgment for the nature of a crime, we may be able to achieve a list of ways in which crimes can be categorized. Herein, we discuss a theory where we combine ML algorithms to act as a database for all recorded crimes in terms of category, along with providing visual knowledge of the surroundings through computer vision techniques, and using the knowledge of such data, we may predict a crime before it occurs.

## Present technological used in crime detection and prediction

Crime forecasting refers to the basic process of predicting crimes before they occur. Tools are needed to predict a crime before it occurs. Currently, there are tools used by police to assist in specific tasks such as listening in on a suspect’s phone call or using a body cam to record some unusual illegal activity. Below we list some such tools to better understand where they might stand with additional technological assistance.

One good way of tracking phones is through the use of a stingray [35], which is a new frontier in police surveillance and can be used to pinpoint a cellphone location by mimicking cellphone towers and broadcasting the signals to trick cellphones within the vicinity to transmit their location and other information. An argument against the usage of stingrays in the United States is that it violates the fourth amendment. This technology is used in 23 states and in the district of Columbia. In ref. [36], the authors provide insight on how this is more than just a surveillance system, raising concerns about privacy violations. In addition, the Federal Communicatons Commission became involved and ultimately urged the manufacturer to meet two conditions in exchange for a grant: (1) “The marketing and sale of these devices shall be limited to federal, state, local public safety and law enforcement officials only” and (2) “State and local law enforcement agencies must advance coordinate with the FBI the acquisition and use of the equipment authorized under this authorization.” Although its use is worthwhile, its implementation remains extremely controversial.

A very popular method that has been in practice since the inception of surveillance is “the stakeout”. A stakeout is the most frequently practiced surveillance technique among police officers and is used to gather information on all types of suspects. In ref. [37], the authors discuss the importance of a stakeout by stating that police officers witness an extensive range of events about which they are required to write a report. Such criminal acts are observed during stakeouts or patrols; observations of weapons, drugs, and other evidence during house searches; and descriptions of their own behavior and that of the suspect during arrest. Stakeouts are extremely useful, and are considered 100% reliable, with the police themselves observing the notable proceedings. However, are they actually 100% accurate? All officers are humans, and all humans are subject to fatigue. The major objective of a stakeout is to observe wrongful activities. Is there a tool that can substitute its use? We will discuss this point herein.

Another way to conduct surveillance is by using drones, which help in various fields such as mapping cities, chasing suspects, investigating crime scenes and accidents, traffic management and flow, and search and rescue after a disaster. In ref. [38], legal issues regarding the use of drones and airspace distribution problems are described. Legal issues include the privacy concerns raised by the public, with the police gaining increasing power and authority. Airspace distribution raises concerns about how high a drone is allowed to go.

Other surveillance methods include face recognition, license plate recognition, and body cams. In ref. [39], the authors indicated that facial recognition can be used to obtain the profile of suspects and analyze it from different databases to obtain more information. Similarly, a license plate reader can be used to access data about a car possibly involved in a crime. They may even use body cams to see more than what the human eye can see, meaning that the reader observes everything a police officer sees and records it. Normally, when we see an object, we cannot recollect the complete image of it. In ref. [40], the impact of body cams was studied in terms of officer misconduct and domestic violence when the police are making an arrest. Body cams are thus being worn by patrol officers. In ref. [41], the authors also mentioned how protection against wrongful police practices is provided. However, the use of body cams does not stop here, as other primary reasons for having a body camera on at all times is to record the happenings in front of the wearer in hopes of record useful events during daily activities or during important operations.

Although each of these methods is effective, one point they share in common is that they all work individually, and while the police can use any of these approaches individually or concurrently, having a machine that is able to incorporate the positive aspects of all of these technologies would be highly beneficial.

## ML techniques used in crime prediction

In ref. [42], a comparative study was carried out between violent crime patterns from the Communities and Crime Unnormalized Dataset versus actual crime statistical data using the open source data mining software Waikato Environment for Knowledge Analysis (WEKA). Three algorithms, namely, linear regression, additive regression, and decision stump, were implemented using the same finite set of features on communities and actual crime datasets. Test samples were randomly selected. The linear regression algorithm could handle randomness to a certain extent in the test samples and thus proved to be the best among all three selected algorithms. The scope of the project was to prove the efficiency and accuracy of ML algorithms in predicting violent crime patterns and other applications, such as determining criminal hotspots, creating criminal profiles, and learning criminal trends.

When considering WEKA [43], the integration of a new graphical interface called Knowledge Flow is possible, which can be used as a substitute for Internet Explorer. IT provides a more concentrated view of data mining in association with the process orientation, in which individual learning components (represented by java beans) are used graphically to show a certain flow of information. The authors then describe another graphical interface called an experimenter, which as the name suggests, is designed to compare the performance of multiple learning schemes on multiple data sets.

In ref. [34], the potential of applying a predictive analysis of crime forecasting in an urban context is studied. Three types of crime, namely, home burglary, street robbery, and battery, were aggregated into grids of 200 m × 250 m and retrospectively analyzed. Based on the crime data of the previous 3 years, an ensemble model was applied to synthesize the results of logistic regression and neural network models in order to obtain fortnightly and monthly predictions for the year 2014. The predictions were evaluated based on the direct hit rate, precision, and prediction index. The results of the fortnightly predictions indicate that by applying a predictive analysis methodology to the data, it is possible to obtain accurate predictions. They concluded that the results can be improved remarkably by comparing the fortnightly predictions with the monthly predictions with a separation between day and night.

In ref. [44], crime predictions were investigated based on ML. Crime data of the last 15 years in Vancouver (Canada) were analyzed for prediction. This machine-learning-based crime analysis involves the collection of data, data classification, identification of patterns, prediction, and visualization. K-nearest neighbor (KNN) and boosted decision tree algorithms were also implemented to analyze the crime dataset. In their study, a total of 560,000 crime datasets between 2003 and 2018 were analyzed, and crime prediction with an accuracy of between 39% and 44% was obtained by predicting the crime using ML algorithms. The accuracy was low as a prediction model, but the authors concluded that the accuracy can be increased or improved by tuning both the algorithms and crime data for specific applications.

In ref. [45], a ML approach is presented for the prediction of crime-related statistics in Philadelphia, United States. The problem was divided into three parts: determining whether the crime occurs, occurrence of crime and most likely crime. Algorithms such as logistic regression, KNN, ordinal regression, and tree methods were used to train the datasets to obtain detailed quantitative crime predictions with greater significance. They also presented a map for crime prediction with different crime categories in different areas of Philadelphia for a particular time period with different colors indicating each type of crime. Different types of crimes ranging from assaults to cyber fraud were included to match the general pattern of crime in Philadelphia for a particular interval of time. Their algorithm was able to predict whether a crime will occur with an astonishing 69% accuracy, as well as the number of crimes ranging from 1 to 32 with 47% accuracy.

In ref. [46], the authors analyzed a dataset consisting of several crimes and predicted the type of crime that may occur in the near future depending on various conditions. ML and data science techniques were used for crime prediction in a crime dataset from Chicago, United States. The crime dataset consists of information such as the crime location description, type of crime, date, time, and precise location coordinates. Different combinations of models, such as KNN classification, logistic regression, decision trees, random forest, a support vector machine (SVM), and Bayesian methods were tested, and the most accurate model was used for training. The KNN classification proved to be the best with an accuracy of approximately 0.787. They also used different graphs that helped in understanding the various characteristics of the crime dataset of Chicago. The main purpose of this paper is to provide an idea of how ML can be used by law enforcement agencies to predict, detect, and solve crime at a much better rate, which results in a reduction in crime.

**Fig. 1:**
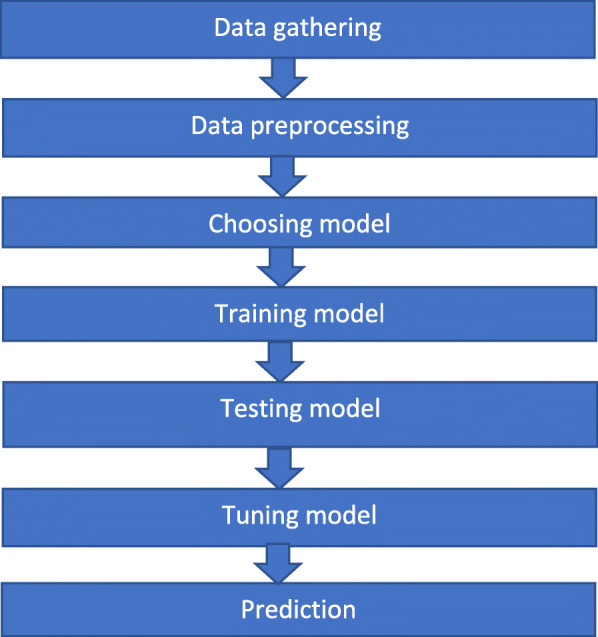
Dataflow diagram

In ref. [47], a graphical user interface-based prediction of crime rates using a ML approach is presented. The main focus of this study was to investigate machine-learning-based techniques with the best accuracy in predicting crime rates and explore its applicability with particular importance to the dataset. Supervised ML techniques were used to analyze the dataset to carry out data validation, data cleaning, and data visualization on the given dataset. The results of the different supervised ML algorithms were compared to predict the results. The proposed system consists of data collection, data preprocessing, construction of a predictive model, dataset training, dataset testing, and a comparison of algorithms, as shown in Fig. 1. The aim of this study is to prove the effectiveness and accuracy of a ML algorithm for predicting violent crimes.

In ref. [48], a feature-level data fusion method based on a deep neural network (DNN) is proposed to accurately predict crime occurrence by efficiently fusing multi-model data from several domains with environmental context information. The dataset consists of data from an online database of crime statistics from Chicago, demographic and meteorological data, and images. Crime prediction methods utilize several ML techniques, including a regression analysis, kernel density estimation (KDE), and SVM. Their approach mainly consisted of three phases: collection of data, analysis of the relationship between crime incidents and collected data using a statistical approach, and lastly, accurate prediction of crime occurrences. The DNN model consists of spatial features, temporal features, and environmental context. The SVM and KDE models had accuracies of 67.01% and 66.33%, respectively, whereas the proposed DNN model had an astonishing accuracy of 84.25%. The experimental results showed that the proposed DNN model was more accurate in predicting crime occurrences than the other prediction models.

**Fig. 2:**
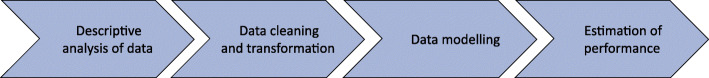
Functionality of proposed approach

In ref. [49], the authors mainly focused on the analysis and design of ML algorithms to reduce crime rates in India. ML techniques were applied to a large set of data to determine the pattern relations between them. The research was mainly based on providing a prediction of crime that might occur based on the occurrence of previous crime locations, as shown in Fig. 2. Techniques such as Bayesian neural networks, the Levenberg Marquardt algorithm, and a scaled algorithm were used to analyze and interpret the data, among which the scaled algorithm gave the best result in comparison with the other two techniques. A statistical analysis based on the correlation, analysis of variance, and graphs proved that with the help of the scaled algorithm, the crime rate can be reduced by 78%, implying an accuracy of 0.78.

In ref. [50], a system is proposed that predicts crime by analyzing a dataset containing records of previously committed crimes and their patterns. The proposed system works mainly on two ML algorithms: a decision tree and KNN. Techniques such as the random forest algorithm and Adaptive Boosting were used to increase the accuracy of the prediction model. To obtain better results for the model, the crimes were divided into frequent and rare classes. The frequent class consisted of the most frequent crimes, whereas the rare class consisted of the least frequent crimes. The proposed system was fed with criminal activity data for a 12-year period in San Francisco, United States. Using undersampling and oversampling methods along with the random forest algorithm, the accuracy was surprisingly increased to 99.16%.

In ref. [51], a detailed study on crime classification and prediction using ML and deep learning architectures is presented. Certain ML methodologies, such as random forest, naïve Bayes, and an SVM have been used in the literature to predict the number of crimes and hotspot prediction. Deep learning is a ML approach that can overcome the limitations of some machine-learning methodologies by extracting the features from the raw data. This paper presents three fundamental deep learning configurations for crime prediction: (1) spatial and temporal patterns, (2) temporal and spatial patterns, and (3) spatial and temporal patterns in parallel. Moreover, the proposed model was compared with 10 state-of-the-art algorithms on 5 different crime prediction datasets with more than 10 years of crime data.

In ref. [52], a big data and ML technique for behavior analysis and crime prediction is presented. This paper discusses the tracking of information using big data, different data collection approaches, and the last phase of crime prediction using ML techniques based on data collection and analysis. A predictive analysis was conducted through ML using RapidMiner by processing historical crime patterns. The research was mainly conducted in four phases: data collection, data preparation, data analysis, and data visualization. It was concluded that big data is a suitable framework for analyzing crime data because it can provide a high throughput and fault tolerance, analyze extremely large datasets, and generate reliable results, whereas the ML based naïve Bayes algorithm can achieve better predictions using the available datasets.

In ref. [53], various data mining and ML technologies used in criminal investigations are demonstrated. The contribution of this study is highlighting the methodologies used in crime data analytics. Various ML methods, such as a KNN, SVM, naïve Bayes, and clustering, were used for the classification, understanding, and analysis of datasets based on predefined conditions. By understanding and analyzing the data available in the crime record, the type of crime and the hotspot of future criminal activities can be determined. The proposed model was designed to perform various operations such as feature selection, clustering, analysis, prediction, and evaluation of the given datasets. This research proves the necessity of ML techniques for predicting and analyzing criminal activities.

In ref. [54], the authors incorporated the concept of a grid-based crime prediction model and established a range of spatial-temporal features based on 84 types of geographic locations for a city in Taiwan. The concept uses ML algorithms to learn the patterns and predict crime for the following month for each grid. Among the many ML methods applied, the best model was found to be a DNN. The main contribution of this study is the use of the most recent ML techniques, including the concept of feature learning. In addition, the testing of crime displacement also showed that the proposed model design outperformed the baseline.

In ref. [55], the authors considered the development of a crime prediction model using the decision tree (J48) algorithm. When applied in the context of law enforcement and intelligence analysis, J48 holds the promise of mollifying crime rates and is considered the most efficient ML algorithm for the prediction of crime data in the related literature. The J48 classifier was developed using the WEKA tool kit and later trained on a preprocessed crime dataset. The experimental results of the J48 algorithm predicted the unknown category of crime data with an accuracy of 94.25287%. With such high accuracy, it is fair to count on the system for future crime predictions.

## Comparative study of different forecasting methods

First, in refs. [56, 57], the authors predicted crime using the KNNs algorithm in the years 2014 and 2013, respectively. Sun et al. [56] proved that a higher crime prediction accuracy can be obtained by combining the grey correlation analysis based on new weighted KNN (GBWKNN) filling algorithm with the KNN classification algorithm. Using the proposed algorithm, we were able to obtain an accuracy of approximately 67%. By contrast, Shojaee et al. [57] divided crime data into two parts, namely, critical and non-critical, and applied a simple KNN algorithm. They achieved an astonishing accuracy of approximately 87%.

Second, in refs. [58, 59], crime is predicted using a decision tree algorithm for the years 2015 and 2013, respectively. In their study, Obuandike et al. [58] used the ZeroR algorithm along with a decision tree but failed to achieve an accuracy of above 60%. In addition, Iqbal et al. [59] achieved a stunning accuracy of 84% using a decision tree algorithm. In both cases, however, a small change in the data could lead to a large change in the structure.

Third, in refs. [60, 61], a novel crime detection technique called naïve Bayes was implemented for crime prediction and analysis. Jangra and Kalsi [60] achieved an astounding crime prediction accuracy of 87%, but could not apply their approach to datasets with a large number of features. By contrast, Wibowo and Oesman [61] achieved an accuracy of only 66% in predicting crimes and failed to consider the computational speed, robustness, and scalability.

Below, we summarize the above comparison and add other models to further illustrate this comparative study and the accuracy of some frequently used models (Table 1).

**Table 1:** Performance analysis of forecasting methods

No.	Forecasting method	Accuracy	Limitations	Observation	References
1	Decision tree (J48)	59.15%	Took more time of 0.76 s to build model compared to 0.09 s of other models.	They took J48 naïve Bayesian and ZeroR and compared them by running tests.	[58]
2	KNN (K = 5)	66.6939%	Data filling algorithms needs to be added to increase the accuracy.	In their research they try to prove that higher accuracy can be achieved if GBWKNN filling algorithm and KNN classification algorithm is combined.	[56]
3	KNN (K = 10)	87.03%	Naïve Bayesian has slightly higher accuracy.	They essentially divided data into critical and non-critical and then compared it in 5 classification algorithms and noted that naïve Bayesian, neural networks, and KNN predict better than the SVM and decision tree.	[57]
4	Naïve Bayes classifier	87.00%	Cannot be applied to the dataset having large number of features.	They implemented a novel crime detection naïve Bayes method for crime prediction and analysis.	[60]
5	Decision tree	83.9519%	As they are unstable, a small change in data can lead to a large change in the structure.	They showed that decision tree performed better than the naïve Bayesian with the same crime dataset, using WEKA.	[59]
6	Naïve Bayes	65.59%	Computational speed, robustness, scalability, and interpretability were not taken into consideration.	This paper presented comparative analysis on the accuracy of k-NN, naive Bayescand decision tree algorithms in predicting crimes and criminal actions.	[61]
7	Autoregressive integrated moving average (ARIMA)	The mean absolute error and standard deviation of the model are (1) Test average test standard deviation is 0.0867 ± 0.0293; (2) Training average training standard deviation is 0.0413 ± 0.0084.	They have described this model as quite complex compared to others.	This paper is about the cons of fuzzy cognitive maps with respect to time series prediction. The ARIMA uses the auto-correlation parameters.	[62]
8	Regression model	They first took 10 crimes per month the expected forecast for absolute percent error (APE) was 42%. When 20 crimes were taken the expected forecast APE was 28%, and at 25 crimes per month the expected forecast APE was 25%. After 30 crimes they 13.5% error.	This research date backs 20 years.	They aim at predicting crimes 30 days ahead. They conduct the experiment in Pittsburgh.	[63]
9	SVM	Over 10 months of experiment its accuracy was 84.37%.	The challenge that they indicated that we may face in the future could be to locate the best point at which spatial knowledge is available.	They compare different model to analyze which has the best chance at predicting hotspots.	[64]
10	Random Forrest Regressor	Ninety-seven percent accuracy in predicting crimes	They got this high accuracy in previous recorded crimes to actually predict crimes in real life will be a challenge.	They had divided their data into 2 parts 80% of it was used to train the model and the rest 20% was used to test the model in this the model achieved the score of 90%.	[65]

## Computer vision models combined with machine and deep learning techniques

In ref. [66], the study focused on three main questions. First, the authors question whether computer vision algorithms actually work. They stated that the accuracy of the prediction is 90% over fewer complex datasets, but the accuracy drops to 60% over complex datasets. Another concern we need to focus on is reducing the storage and computational costs. Second, they question whether it is effective for policing. They determined that a distinct activity detection is difficult, and pinpointed a key component, the Public Safety Visual Analytics Workstation, which includes many capabilities ranging from detection and localization of objects in camera feeds to labeling actions and events associated with training data, and allowing query-based searches for specific events in videos. By doing so, they aim to view every event as a computer-vision trained, recognized, and labeled event. The third and final question they ask is whether computer vision impacts the criminal justice system. The answer to this from their end is quite optimistic to say the least, although they wish to implement computer vision alone, which we suspect is unsatisfactory.

In ref. [67], a framework for multi-camera video surveillance is presented. The framework is designed so efficiently that it performs all three major activities of a typical police “stake-out”, i.e., detection, representation, and recognition. The detection part mixes video streams from multiple cameras to efficiently and reliably extract motion trajectories from videos. The representation helps in concluding the raw trajectory data to construct hierarchical, invariant, and content-rich descriptions of motion events. Finally, the recognition part deals with event classification (such as robbery and possibly murder and molestation, among others) and identification of the data descriptors. For an effective recognition, they developed a sequence-alignment kernel function to perform sequence data learning to identify suspicious/possible crime events.

In ref. [68], a method is suggested for identifying people for surveillance with the help of a new feature called soft biometry, which includes a person’s height, built, skin tone, shirt and trouser color, motion pattern, and trajectory history to identify and track passengers, which further helps in predicting crime activities. They have gone further and discussed some absurd human error incidents that have resulted in the perpetrators getting away. They also conducted experiments, the results of which were quite astounding. In one case, the camera catches people giving piggyback rides in more than one frame of a single shot video. The second scenario shows the camera’s ability to distinguish between airport guards and passengers.

In ref. [69], the authors discussed automated visual surveillance in a realistic scenario and used Knight, which is a multiple camera surveillance and monitoring system. Their major targets were to analyze the detection, tracking, and classification performances. The detection, tracking, and classification accuracies were 97.4%, 96.7%, and 88%, respectively. The authors also pointed to the major difficulties of illumination changes, camouflage, uninteresting moving objects, and shadows. This research again proves the reliability of computer vision models.

It is well known that an ideal scenario for a camera to achieve a perfect resolution is not possible. In ref. [70], security surveillance systems often produce poor-quality video, which could be a hurdle in gathering forensic evidence. They examined the ability of subjects to identify targeted individuals captured by a commercially available video security device. In the first experiment, subjects personally familiar with the targets performed extremely well at identifying them, whereas subjects unfamiliar with the targets performed quite poorly. Although these results might not seem to be very conclusive and efficient, police officers with experience in forensic identification performed as poorly as other subjects unfamiliar with the targets. In the second experiment, they asked how familiar subjects could perform so well, and then used the same video device edited clips to obscure the head, body, or gait of the targets. Hiding the body or gait produced a small decrease in recognition performance. Hiding the target heads had a dramatic effect on the subject’s ability to recognize the targets. This indicates that even if the quality of the video is low, the head the target was seen and recognized.

In ref. [71], an automatic number plate recognition (ANPR) model is proposed. The authors described it as an “image processing innovation”. The ANPR system consists of the following steps: (1) vehicle image capture, (2) preprocessing, (3) number plate extraction, (4) character segmentation, and (5) character recognition. Before the main image processing, a pre-processing of the captured image is conducted, which includes converting the red, green and blue image into a gray image, clamor evacuation, and border enhancement for brightness. The plate is then separated by judging its size. In character segmentation, the letters and numbers are separated and viewed individually. In character recognition, optical character recognition is applied to a given database.

Although real-time crime forecasting is vital, it is extremely difficult to achieve in practice. No known physical models provide a reasonable approximation with dependable results for such a complex system. In ref. [72], the authors adapted a spatial temporal residual network to well-represented data to predict the distribution of crime in Los Angeles at an hourly scale in neighborhood-sized parcels. These experiments were compared with several existing approaches for prediction, demonstrating the superiority of the proposed model in terms of accuracy. They compared their deep learning approach to ARIMA, KNN, and the historical average. In addition, they presented a ternarization technique to address the concerns of resource consumption for deployment in the real world.

In ref. [73], the authors conducted a significant study on crime prediction and showed the importance of non-crime data. The major objective of this research was taking advantage of DNNs to achieve crime prediction in a fine-grain city partition. They made predictions using Chicago and Portland crime data, which were further augmented with additional datasets covering the weather, census data, and public transportation. In the paper they split each city into grid cells (beats for Chicago and square grid for Portland). The crime numbers are broken into 10 bins, and their model predicts the most likely bin for each spatial region at a daily level. They train these data using increasingly complex neural network structures, including variations that are suited to the spatial and temporal aspects of the crime prediction problem. Using their model, they were able to predict the correct bin for the overall number of crimes with an accuracy of 75.6% for Chicago and 65.3% for Portland. They showed that adding the value of additional non-crime data was an important factor. They found that days with higher amounts of precipitation and snow decreased the accuracy of the model slightly. Then, considering the impact of transportation, the bus routes and train routes were presented within their beats, and it was shown that the beat containing a train station is on average 1.2% higher than its neighboring beats. The accuracy of a beat that contained one or more train lines passing through it was 0.5% more accurate than its neighboring beats.

In ref. [74], the authors taught a system how to monitor traffic and identify vehicles at night. They used the bright spots of the headlights and tail lights to identify an object first as a vehicle, and the bright light is extracted with a segmentation process, and then processed by a spatial clustering and tracking procedure that locates and analyzes the spatial and temporal features of the vehicle light. They also conducted an experiment in which, for a span of 20 min, the detection scores for cars and bikes were 98.79% and 96.84%, respectively. In another part of the test, they conducted the same test under the same conditions for 50 min, and the detection scores for cars and bikes were 97.58% and 98.48%, respectively. It is good for machines to be built at such a beginning level. This technology can also be used to conduct surveillance at night.

In ref. [75], an important approach for human motion analysis is discussed. The author mentions that human motion analysis is difficult because appearances are extremely variable, and thus stresses that focusing on marker-less vision-based human motion analysis has the potential to provide a non-obtrusive solution for the evaluation of body poses. The author claims that this technology can have vast applications such as surveillance, human-computer interaction, and automatic annotation, and will thus benefit from a robust solution. In this paper, the characteristics of human motion analysis are discussed. We divide the analysis part into two aspects, modeling and an estimation phase. The modeling phase includes the construction of the likelihood function [including the camera model, image descriptors, human body model and matching function, and (physical) constraints], and the estimation phase is concerned with finding the most likely pose given the likelihood (function result) of the surface. We discuss the model-free approaches separately.

In ref. [76], the authors provided insight into how we can achieve crime mapping using satellites. The need for manual data collection for mapping is costly and time consuming. By contrast, satellite imagery is becoming a great alternative. In this paper, they attempted to investigate the use of deep learning to predict crime rates directly from raw satellite imagery. They trained a deep convolutional neural network (CNN) on satellite images obtained from over 1 million crime-incident reports (15 years of data) collected by the Chicago Police Department. The best performing model predicted crime rates from raw satellite imagery with an astounding accuracy of 79%. To make their research more thorough, they conducted a test for reusability, and used the tested and learned Chicago models for prediction in the cities of Denver and San Francisco. Compared to maps made from years of data collected by the corresponding police departments, their maps have an accuracy of 72% and 70%, respectively. They concluded the following: (1) Visual features contained in satellite imagery can be successfully used as a proxy indicator of crime rates; (2) ConvNets are capable of learning models for crime rate prediction from satellite imagery; (3) Once deep models are used and learned, they can be reused across different cities.

In ref. [77], the authors suggested an extremely intriguing research approach in which they claim to prove that looking beyond what is visible is to infer meaning to what is viewed from an image. They even conducted an interesting study on determining where a McDonalds could be located simply from photographs, and provided the possibility of predicting crime. They compared the human accuracy on this task, which was 59.6%, and the accuracy of using gradient-based features, which was 72.5%, with a chance performance (a chance performance is what you would obtain if you performed at random) of only 50%. This indicates the presence of some visual cues that are not easily spotted by an average human, but are able to be spotted by a machine, thus enables us to judge whether an area is safe. The authors indicated that numerous factors are often associated with our intuition, which we use to avoid certain areas because they may seem “shady” or “unsafe”.

In ref. [78], the authors describe in two parts how close we are to achieving a fully automated surveillance system. The first part views the possibility of surveillance in a real-world scenario where the installation of systems and maintenance of systems are in question. The second part considers the implementation of computer vision models and algorithms for behavior modeling and event detection. They concluded that the complete scenario is under discussion, and therefore many people are conducting research and obtaining results. However, as we look closely, we can see that reliable results are possible only in certain aspects, while other areas are still in the development process, such as obtaining information on cars and their owners as well as accurately understanding the behavior of a possible suspect.

Many times during criminal activities, convicts use hand gestures to signal messages to each other. In ref. [79], research on hand gesture recognition was conducted using computer vision models. Their application architecture is of extremely high quality and is easy to understand. They begin by capturing images, and then try detecting a hand in the background. They apply either computer aided manufacturing or different procedure in which they first convert a picture into gray scale, after which they set the image return on investment, and then find and extract the biggest contour. They then determine the convex hull of the contour to try and find an orientation around the bounded rectangle, and finally interpret the gesture and convert it into a meaningful command.

Crime hotspots or areas with high crime intensity are places where the future possibility of a crime exists along with the possibility of spotting a criminal. In ref. [80], the authors conducted research on forecasting crime hotspots. They used Google Tensor Flow to implement their model and evaluated three options for the recurrent neural network (RNN) architecture: accuracy, precision, and recall. The focus is on achieving a larger value to prove that the approach has a better performance. The gated recurrent unit (GRU) and long short-term memory (LSTM) versions obtained similar performance levels with an accuracy of 81.5%, precision of 86%–87%, recall of 75%, and F1-score of 0.8. Both perform much better than the traditional RNN version. Based on the area under the ROC curve (AUC) performance observations, the GRU version was 2% better than the RNN version. The LSTM version achieved the best AUC score, which was improved by 3% over the GRU version.

In ref. [81], a spatiotemporal crime network (STCN) is proposed that applies a CNN for predicting crime before it occurs. The authors evaluated the STCN using 311 felony datasets from New York from 2010 to 2015. The results were extremely impressive, with the STCN achieving an F1-score of 88% and an AUC of 92%, which confirmed that it exceeded the performance of the four baselines. Their proposed model achieved the best performance in terms of both F1 and AUC, which remained better than those of the other baselines even when the time window reached 100. This study provides evidence that the system can function well even in a metropolitan area.

## Proposed idea

After finding and understanding various distinct methods used by the police for surveillance purposes, we determined the importance of each method. Each surveillance method can perform well on its own and produce satisfactory results, although for only one specific characteristic, that is, if we use a Sting Ray, it can help us only when the suspect is using a phone, which should be switched on. Thus, it is only useful when the information regarding the stake out location is correct. Based on this information, we can see how the ever-evolving technology has yet again produced a smart way to conduct surveillance. The introduction of deep learning, ML, and computer vision techniques has provided us with a new perspective on ways to conduct surveillance. This is an intelligent approach to surveillance because it tries to mimic a human approach, but it does so 24 h a day, 365 days a year, and once it has been taught how to do things it does them in the same manner repeatedly.

Although we have discussed the aspects that ML and computer vision can achieve, but what are these aspects essentially? This brings us to the main point of our paper discussion, i.e., our proposed idea, which is to combine the point aspects of Sting Ray, body cams, facial recognition, number plate recognition, and stakeouts. New features iclude core analytics, neural networks, heuristic engines, recursion processors, Bayesian networks, data acquisition, cryptographic algorithms, document processors, computational linguistics, voiceprint identification, natural language processing, gait analysis, biometric recognition, pattern mining, intel interpretation, threat detection, threat classification. The new features are completely computer dependent and hence require human interaction for development; however, once developed, it functions without human interaction and frees humans for other tasks. Let us understand the use of each function.

1. Core analytics: This includes having knowledge of a variety of statistical techniques, and by using this knowledge, predict future outcomes, which in our case are anything from behavioral instincts to looting a store in the near future.
2. Neural networks: This is a concept consisting of a large number of algorithms that help in finding the relation between data by acting similar to a human brain, mimicking biological nerve cells and hence trying to think on its own, thus understanding or even predicting a crime scene.
3. Heuristic engines: These are engines with data regarding antiviruses, and thus knowledge about viruses, increasing the safety of our system as it identifies the type of threat and eliminates it using known antiviruses.
4. Cryptographic algorithms: Such algorithms are used in two parts. First, they privately encode the known confidential criminal data. Second, they are used to keep the newly discovered potential crime data encrypted.
5. Recursion processors: These are used to apply the functions of our machine repeatedly to make sure they continuously work and never break the surveillance of the machine.
6. Bayesian networks: These are probabilistic acyclic graphical models that can be used for a variety of purposes such as prediction, anomaly detection, diagnostics, automated insight, reasoning, time series prediction, and decision making under uncertainty.
7. Data acquisition: This might be the most important part because our system has to possess the knowledge of previous crimes and learn from them to predict future possible criminal events.
8. Document processors: These are used after the data collection, primarily for going through, organizing, analyzing, and learning from the data.
9. Computer linguistics: Using algorithms and learning models, this method is attempting to give a computer the ability to understand human spoken language, which would be ground breaking, allowing a machine to not only identify a human but also understands what the human is saying.
10. Natural language processor: This is also used by computers to better understand human linguistics.
11. Voice print identification: This is an interesting application, which tries to distinguish one person’s voice from another, making it even more recognizable and identifiable. It identifies a target with the help of certain characteristics, such as the configuration of the speaker’s mouth and throat, which can be expressed as a mathematical formula.
12. Gait analysis: This will be used to study human motion, understanding posture while walking. It will be used to better understand the normal pace of a person and thus judge an abnormal pace.
13. Bio metric identification: This is used to identify individuals by their face, or if possible, identify them by their thumb print stored in few different databases.
14. Pattern mining: This is a subset of data mining and helps in observing patterns among routine activities. The use of this technology will help us identify if a person is seen an usual number of times behind a pharmacy window at particular time, allowing the machine to alert the authorities.
15. Intel interpretation: This is also used to make sense of the information gathered, and will include almost all features mentioned above, combining the results of each and making a final meaningful prediction.
16. Threat detection: A threat will be detected if during the intel processing a certain number of check boxes predefined when making the system are ticked.
17. Threat classification: As soon as a threat is detected, it is classified, and the threat can then be categorized into criminal case levels, including burglary, murder, or a possible terrorist attack; thus, based on the time line, near or distant future threats might be predictable.

Combining all of these features, we aim to produce software that has the capability of becoming a universal police officer, having eyes and ears everywhere. Obviously, we tend to use the CCTVs in urban areas during a preliminary round to see the functioning of such software in a real-world scenario. The idea is to train and make the software learn all previously recorded crimes whose footages are available (at least 5000 cases for optimum results), through supervised learning, unsupervised learning, semi-supervised learning, and reinforcement learning to help it to understand what a crime actually is. Thus, it will achieve a better understanding of criminality and can answer how crimes happen, as well as why and where. We do not propose simply making a world-class model to predict crimes, we also suggest making it understand previous crimes to better judge and therefore better predict them.

We aim to use this type of technology on two fronts: first and most importantly, for predicting crimes before they happen, followed by a thorough analysis of a crime scene allowing the system to possibly identify aspects that even a human eye can miss.

The most interesting cutting-edge and evolutionary idea that we believe should be incorporated is the use of scenario simulations. After analyzing the scene and using the 17 main characteristics mentioned above, the software should run at least 50 simulations of the present scenario presented in front of it, which will be assisted by previously learned crime recordings. The simulation will help the software in asserting the threat level and then accordingly recommend a course of action or alert police officials.

**Fig. 3:**
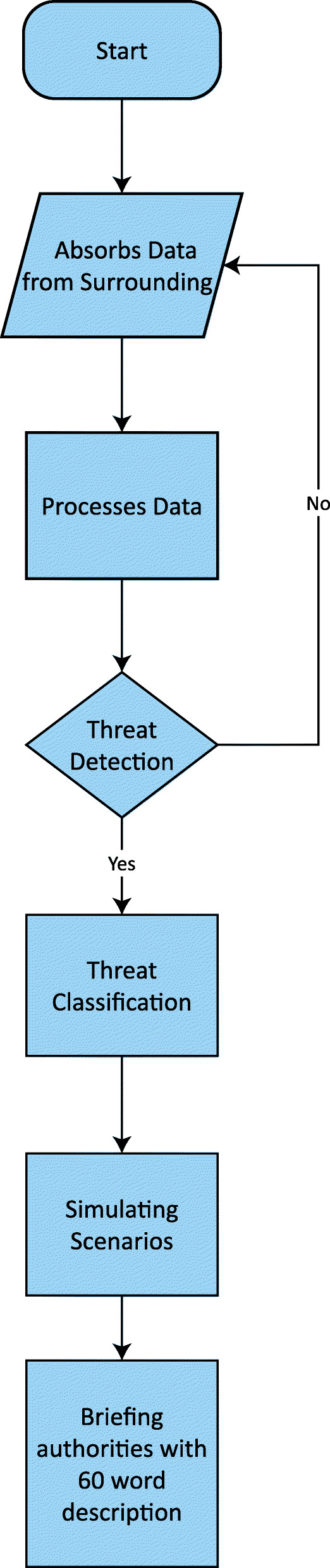
Flowchart of our proposed model. The data are absorbed from the surrounding with the help of cameras and microphones. If the system depicts an activity as suspicious, it gathers more intel allowing the facial algorithms to match against a big database such as a Social Security Number or Aadhaar card database. When it detects a threat, it also classifies it into categories such as the nature of the crime and time span within which it is possible to take place. With all the gathered intel and all the necessary details of the possible crime, it alerts the respective authority with a 60-word synopsis to give them a brief idea, allowing law enforcement agencies to take action accordingly

To visualize a possible scenario where we are able to invent such software, we prepared a flow chart (Fig. 3) to better understand the complete process.

## Challenges

Although this paper has been implemented with high accuracy and detailed research, there are certain challenges that can pose a problem in the future. First, the correct and complete building of the whole system has to be done in the near future, allowing its implementation to take place immediately and properly. Furthermore, the implementation itself is a significant concern, as such technologies cannot be directly implemented in the open world. The system must first be tested in a small part of a metropolitan area, and only then with constant improvements (revisions of the first model) can its usage be scaled up. Hence, the challenges are more of a help in perfecting the model and thus gradually providing a perfect model that can be applied to the real world. Moreover, there are a few hurdles in the technological aspects of the model, as the size of the learning data will be enormous, and thus processing it will take days and maybe even weeks. Although these are challenges that need to be addressed, they are aspects that a collective team of experts can overcome after due diligence, and if so, the end product will be worth the hard work and persistence.

## Future scope

This paper presented the techniques and methods that can be used to predict crime and help law agencies. The scope of using different methods for crime prediction and prevention can change the scenario of law enforcement agencies. Using a combination of ML and computer vision can substantially impact the overall functionality of law enforcement agencies. In the near future, by combining ML and computer vision, along with security equipment such as surveillance cameras and spotting scopes, a machine can learn the pattern of previous crimes, understand what crime actually is, and predict future crimes accurately without human intervention. A possible automation would be to create a system that can predict and anticipate the zones of crime hotspots in a city. Law enforcement agencies can be warned and prevent crime from occurring by implementing more surveillance within the prediction zone. This complete automation can overcome the drawbacks of the current system, and law enforcement agencies can depend more on these techniques in the near future. Designing a machine to anticipate and identify patterns of such crimes will be the starting point of our future study. Although the current systems have a large impact on crime prevention, this could be the next big approach and bring about a revolutionary change in the crime rate, prediction, detection, and prevention, i.e., a “universal police officer”.

## Conclusions

Predicting crimes before they happen is simple to understand, but it takes a lot more than understanding the concept to make it a reality. This paper was written to assist researchers aiming to make crime prediction a reality and implement such advanced technology in real life. Although police do include the use of new technologies such as Sting Rays and facial recognition every few years, the implementation of such software can fundamentally change the way police work, in a much better way. This paper outlined a framework envisaging how the aspects of machine and deep learning, along with computer vision, can help create a system that is much more helpful to the police. Our proposed system has a collection of technologies that will perform everything from monitoring crime hotspots to recognizing people from their voice notes. The first difficulty faced will be to actually make this system, followed by problems such as its implementation and use, among others. However, all of these problems are solvable, and we can also benefit from a security system that monitors the entire city around-the-clock. In other words, to visualize a world where we incorporate such a system into a police force, tips or leads that much more reliable can be achieved and perhaps crime can be eradicated at a much faster rate.

## Data Availability

All relevant data and material are presented in the main paper.

## Abbreviations

ML: Machine learning
NP: Nondeterministic polynomial
WEKA: Waikato Environment for Knowledge Analysis
KNN: K-nearest neighbor
ANPR: Automatic number plate recognition
DNN: Deep neural network
KDE: Kernel density estimation
SVM: Support vector machine
GBWKNN: Grey correlation analysis based on new weighted KNN
ARIMA: Autoregressive integrated moving average
STCN: Spatiotemporal crime network
CNN: Convolutional neural network
AUC: Area under the ROC curve
RNN: Recurrent neural network
GRU: Gated recurrent unit
LSTM: Long short-term memory
APE: Absolute percent error
